# Cerebral Microdialysate Metabolite Monitoring using Mid-infrared Spectroscopy

**DOI:** 10.1021/acs.analchem.1c01149

**Published:** 2021-08-25

**Authors:** Farah C. Alimagham, Dan Hutter, Núria Marco-García, Emma Gould, Victoria H. Highland, Anna Huefner, Susan Giorgi-Coll, Monica J. Killen, Agnieszka P. Zakrzewska, Stephen R. Elliott, Keri L. H. Carpenter, Peter J. Hutchinson, Tanya Hutter

**Affiliations:** † Department of Chemistry, University of Cambridge, Cambridge CB2 1EW, United Kingdom; ‡ Division of Neurosurgery, Department of Clinical Neurosciences, University of Cambridge, Cambridge CB2 0QQ, United Kingdom; § Department of Electrical and Computer Engineering, The University of Texas at Austin, Austin, Texas 78712, United States; ∥ Materials Science and Engineering Program and Texas Materials Institute, University of Texas at Austin, Austin, Texas 78712, United States; ⊥ Walker Department of Mechanical Engineering, The University of Texas at Austin, Austin, Texas 78712, United States

## Abstract

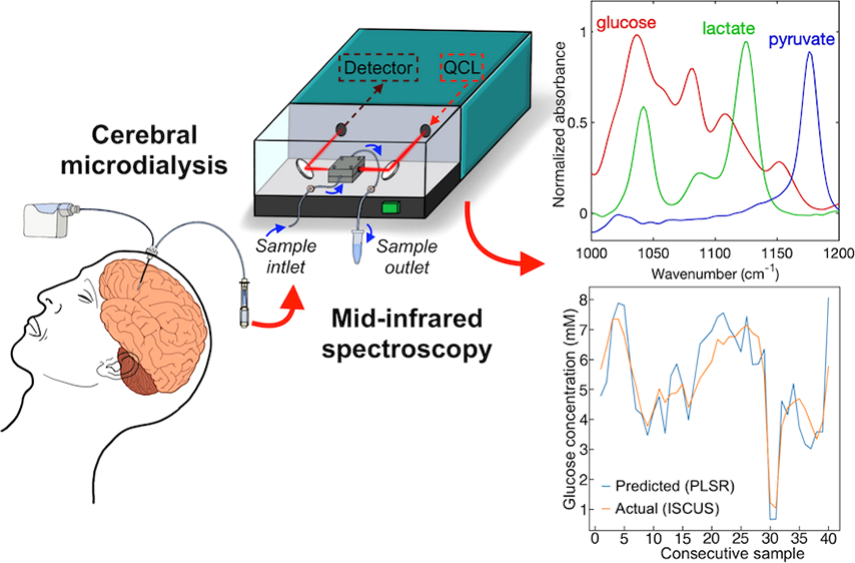

The brains of patients suffering from traumatic brain-injury (TBI) undergo dynamic chemical changes in the days following the initial trauma. Accurate and timely monitoring of these changes is of paramount importance for improved patient outcome. Conventional brain-chemistry monitoring is performed off-line by collecting and manually transferring microdialysis samples to an enzymatic colorimetric bedside analyzer every hour, which detects and quantifies the molecules of interest. However, off-line, hourly monitoring means that any subhourly neurochemical changes, which may be detrimental to patients, go unseen and thus untreated. Mid-infrared (mid-IR) spectroscopy allows rapid, reagent-free, molecular fingerprinting of liquid samples, and can be easily integrated with microfluidics. We used mid-IR transmission spectroscopy to analyze glucose, lactate, and pyruvate, three relevant brain metabolites, in the extracellular brain fluid of two TBI patients, sampled via microdialysis. Detection limits of 0.5, 0.2, and 0.1 mM were achieved for pure glucose, lactate, and pyruvate, respectively, in perfusion fluid using an external cavity-quantum cascade laser (EC-QCL) system with an integrated transmission flow-cell. Microdialysates were collected hourly, then pooled (3–4 h), and measured consecutively using the standard ISCUSflex analyzer and the EC-QCL system. There was a strong correlation between the compound concentrations obtained using the conventional bedside analyzer and the acquired mid-IR absorbance spectra, where a partial-least-squares regression model was implemented to compute concentrations. This study demonstrates the potential utility of mid-IR spectroscopy for continuous, automated, reagent-free, and online monitoring of the dynamic chemical changes in TBI patients, allowing a more timely response to adverse brain metabolism and consequently improving patient outcomes.

## Introduction

Traumatic brain injury (TBI) is the leading cause of death in those aged under 40 years in the developed world, typically resulting from road and sporting accidents, falls, and violence.^1^ In addition to the high mortality, approximately 60% of survivors have significant ongoing deficits.^2^ Following the initial traumatic event, complex changes evolve in the injured brain, which may result in secondary damage in the following hours and days, leading to highly unfavorable outcomes, such as severe disability, vegetative state, or death.^3^ These processes are potentially amenable to intervention, and, therefore, close monitoring is a key element in the management of the injured brain.^4,5^ Among various modalities used to monitor brain physiology are direct monitoring of the intracranial pressure (ICP), brain-tissue oxygen (PbtO_2_), and the cerebral extracellular chemistry.^4,5^ While ICP and PbtO_2_ are both monitored in real-time, allowing a rapid response to any dangerous changes in these markers, brain chemistry is monitored hourly using a technique called cerebral microdialysis. The microdialysis technique used in clinical practice requires the collection of microdialysate into vials and their manual transfer into a bedside analyzer (ISCUSflex, M Dialysis AB, Stockholm, Sweden) every hour.^6^ The fact that microdialysis is limited to hourly readings means that any rapid changes in brain chemistry can be overlooked and opportunities for timely intervention are lost. Moreover, it requires manual transfer of samples and is based on enzymatic colorimetric assays, which require a range of reagents. There is a clinical need for a sensor system, which would allow continuous online monitoring of glucose, lactate, and pyruvate, the three most clinically relevant substances in TBI-patient microdialysate,^6,7^ over several days at the patient bedside, using methods which do not require expensive consumables or manual labor. Based on existing evidence,^8,9^ such a system would be of great value and highly beneficial for clinicians, nurses, and most importantly for favorable TBI-patient outcome. Research has already been carried out toward developing a real-time microdialysate-analyzing sensor,^9−12^ and a new product for real-time microdialysis monitoring, LOKE (M Dialysis AB, Stockholm, Sweden), has recently been developed. However, these studies and developments are mostly based on enzymatic-electrochemical detection, which, despite providing a high sensitivity, has the disadvantages of frequently requiring a number of fresh reagents and consumables, having a relatively complex fabrication process and which also alter/consume the sample of interest.

Spectroscopy techniques, such as fluorescence, Raman, UV–vis and infrared (IR) absorption spectroscopies, provide access to the chemical composition of samples in microliter volumes,^13^ and their suitability depends on the nature of the material in question. Mid-IR absorption spectroscopy offers direct access to the structure of molecules by measuring their fundamental fingerprint vibrational spectra, thus providing rapid, non-destructive and label-free molecular detection.^14^ These features allow the possibility of continuous sample monitoring, and their postutilization for further studies. Fourier-transform IR (FT-IR) spectroscopy is considered the gold standard in mid-IR spectroscopy,^15^ and is a well-established tool for qualitative and quantitative analysis of substances in liquid and gaseous phases.^16−19^ Molecular interferences can be minimized by implementing multivariate-analysis techniques, such as partial-least-squares regression (PLSR).^15,20^ However, FT-IR spectrometers are generally equipped with thermal broadband light sources (e.g., Globar), which emit low-power IR-radiation, restricting transmission path-lengths for analyte measurements in liquids due to strongly absorbing solvents (e.g., water), and consequently limiting achievable sensitivities.^15^ Developments in the last two decades of powerful mid-IR light sources with high spectral power density, namely quantum cascade lasers (QCLs), have led to systems, which surpass conventional FT-IR spectrometers in terms of their performance, and allow significantly improved sensitivity and selectivity.^15,21^ Broadly tunable external cavity (EC) QCLs, in particular, have been extensively implemented for multianalyte detection of physiologically relevant substances in the liquid phase.^15,22,23^

In the present study, we demonstrate the use of mid-IR spectroscopy for the detection, quantification, and monitoring of physiological concentrations of glucose, lactate, and pyruvate in the extracellular brain fluid of TBI patients, using an EC-QCL system with an integrated flow-cell. Statistical analysis was performed on the spectra of each pure compound and the limits of detection and quantification were determined. TBI-patient cerebral microdialysates, collected consecutively from two patients, were measured on both the EC-QCL system and the current clinical “gold-standard” (ISCUSflex) analyzer to test for correlations. A PLSR model was developed using synthetic microdialysis samples to compute the concentration of glucose and lactate in patients’ microdialysates. Here, we demonstrate the first step toward the implementation of continuous, online, label-free, and reagent-free cerebral microdialysis monitoring using mid-IR spectroscopy, which will ultimately allow improved patient management and outcome.

## Materials and Methods

### Standards and Reagents

Pure solutions of glucose, lactate, and pyruvate within their physiological range were prepared by dissolving d-(+)-glucose, sodium l-lactate, and sodium pyruvate (all ≥99%, Sigma-Aldrich, Gillingham, Dorset, UK) in perfusion fluid, a sterile, isotonic fluid especially developed for clinical microdialysis. For in vitro work, a stock solution of perfusion fluid was prepared with the same composition as the perfusion fluid used clinically (CNS Perfusion Fluid, M Dialysis AB, Stockholm, Sweden), containing 147 mM NaCl, 2.7 mM KCl, 1.2 mM CaCl_2_, and 0.85 mM MgCl_2_ dissolved in ultrapure 18.2 MΩ·cm water (Direct-Q5 UV, Millipore), with all compounds purchased from Sigma-Aldrich, and subsequently filtered using a syringe filter (5556-06, 0.22 μm poly(vinylidene difluoride) membrane, PRO-MEM, Essex, UK).

### QCL-IR and FT-IR Operation and Measurements

**Figure 1:**
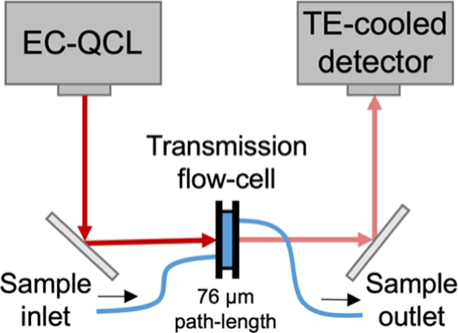
Simplified schematic of the QCL-based mid-IR transmission setup for analysis of aqueous samples.

QCL-IR measurements were performed using a commercially available QCL-IR spectrometer with an integrated flow-cell (ChemDetect analyzer, Daylight Solutions, Inc., San Diego, USA). It is a compact and portable instrument, which allows the continuous analysis and identification of chemical compounds within fluids, and comprises a tunable EC-QCL light source, a microfluidic flow-cell, and a thermoelectrically cooled InAsSb (indium arsenide antimonide) detector, as shown in Figure 1, and with more detail, in Figure S1A.

The emergent QCL beam is directed (via coupling optics and a mirror) into the flow-cell, where it interacts with a flowing sample, and the light is then redirected into the detector, resulting in the absorbance spectrum of the sample. The QCL is broadly tunable within the mid-IR fingerprint region between 982 and 1258 cm^–1^, and sweeps are made with 0.2 cm^–1^ steps and averaged to 2 cm^–1^ resolution. A single sweep is taken in approximately 1 s, but several sweeps can be averaged for an increased signal-to-noise ratio in the final spectrum. A cooling system (UC160-190, Solid State Cooling Systems, USA) was used in conjunction with the ChemDetect analyzer to ensure temperature stability during measurements by recirculating a mixture of chilled water and a liquid coolant (702 Liquid Coolant, Koolance, South Korea) in the bottom plate of the instrument via two rear inlet/outlet connectors. The ChemDetect analyzer is operated with its own ChemDetect software. The flow-cell, shown in Figure S1B, has a small channel volume of ∼1 μL, diamond windows, and an adjustable path-length, through the selection of different poly(tetrafluoroethylene)-gasket thicknesses. In this study, a path-length of 76 μm was used, and each sample was measured three times, obtained by averaging 80 spectra over approximately 5 min. FT-IR measurements were acquired, for comparison, using a conventional FT-IR spectrometer (Spectrum 100 FT-IR, PerkinElmer, USA) with a 50 μm liquid-transmission accessory (Oyster cell and Pearl, Specac, Orpington, UK). Spectra were acquired between 400 and 4500 cm^–1^ via the accumulation of 150 scans with a resolution of 4 cm^–1^. Plain perfusion fluid was used as a background for all measurements. The results are presented between 1000 and 1200 cm^–1^ because glucose, lactate, and pyruvate show unique spectral features in this range,^24^ where a moderately reduced water absorption is observed compared to other regions of the mid-IR. To account for small offsets in the overall sample absorbance, single-point baseline correction was applied to the acquired spectra of pure compounds, where the absorbance value at a specific wavelength (at which there is no absorbance attributed to the sample buffer or the molecule of interest) was subtracted from all wavelengths across the spectrum. For glucose and lactate spectral baseline correction, the point at 1180 cm^–1^ was used, while for pyruvate, the point at 1080 cm^–1^ was used. Relative absorbance peak intensities were determined following baseline correction.

### Statistical Analysis, LOD, and LOQ

One-way analysis of variance (ANOVA) was conducted on each spectral dataset, using the “anova1” function of the Statistics and Machine Learning Toolbox (Matlab R2020b, MathWorks, Inc., Natick, MA, USA), to determine whether any of the mean values of the relative peak intensities were statistically significantly different from one another. A post-hoc multiple-comparison test was subsequently performed, using the “multcompare” function, also included in the Statistics and Machine Learning Toolbox, in order to identify which of the mean values of the relative peak intensity within a dataset were statistically significantly different from one another. The test compares the mean relative peak intensity at a given concentration with those at all other concentrations. This process was repeated for each concentration, so that the mean relative peak intensities at all concentrations were systematically compared with one another. The resulting output was used to determine which concentrations were statistically significantly different from other concentrations, and hence, which concentrations could be detected and differentiated, thus providing a measure of the limit of quantification (LOQ). For comparison, the limit of detection (LOD) and LOQ were also estimated as LOD = 3 × σ/*S* and LOQ = 10 × σ/*S*, where σ is the standard deviation (SD) of the response, which was determined based on the residual SD of the regression line, and *S* is the slope of the calibration curve. All values presented are for single-analyte measurements in perfusion fluid.

### Patient Sample Collection and Pooling

This study was approved by the East of England—Cambridge Central Research Ethics Committee (REC# 17/EE/0321, IRAS# 214601). Informed written consent was obtained from the next-of-kin of each patient. Patients (age over 18 years) with major TBI included in the study underwent monitoring of cerebral chemistry, with collection of the extracellular fluid via microdialysis catheters (M Dialysis 71, M Dialysis AB). The catheters were inserted in the frontal white matter via a cranial access device (Technicam, Newton Abbot, UK), along with the ICP probe, and perfused with CNS perfusion fluid at 0.3 μL min^–1^ using a portable syringe pump (CMA 107 M Dialysis AB, Stockholm, Sweden), with microdialysis collection vials changed hourly. Microdialysate samples were collected and stored in the freezer at −75 °C for subsequent pooling and analysis using the ISCUSflex analyzer and the ChemDetect analyzer for concentrations of glucose, lactate, and pyruvate. The contents of 3 or 4 consecutive sample vials were pooled together to achieve sufficient volume for measurements on both instruments. These were then split equally into two separate vials: one to be measured on the ISCUS and the other to be measured on the ChemDetect. All samples were labeled in consecutive order and immediately frozen at −75 °C until just before the measurements. Consecutive samples were measured for patient 1 (16 samples) and patient 2 (40 samples). Both ISCUS and ChemDetect measurements were carried out simultaneously in order to avoid any discrepancies between identical samples, for example, due to sample evaporation.

### Synthetic Sample Preparation for Multivariate Analysis

To obtain a statistically viable model, a chemically diverse range of synthetic samples were prepared and used for the development of the PLSR model for subsequent use in predicting patient microdialysate concentrations. A rejection sampling algorithm was developed and used to generate a list of 50 samples containing different combinations of glucose, lactate, and pyruvate concentrations within ranges which are typical in TBI-patients' microdialysates.^5^ The full list of 50 samples with the respective concentrations are shown in Table S1 and the histograms in Figure S2 indicate the frequency at which the various concentrations of glucose, lactate, and pyruvate were observed in the generated list of samples. Table 1 summarizes the distribution properties respective of each compound.

**Table 1:** Synthetic Sample Distribution Properties for PLSR Model Development; Mean, SD, and Maximum and Minimum Values for Glucose, Lactate, and Pyruvate

compound	mean (mM)	SD (mM)	max (mM)	min (mM)
glucose	2.34	1.52	5.0	0.02
lactate	4.48	2.07	8.0	0.5
Pyruvate	0.17	0.07	0.30	0.02

### Spectral Preprocessing

Four outliers were determined by visual inspection of the spectra of synthetic samples and were excluded for the generation of the regression model. These were likely caused by bubbles passing through the flow-cell, thus altering the spectral features. The remaining 46 spectra used to generate and test the model are shown in Figure S3A. First-order differencing was performed as a preprocessing step and the spectral range was limited to 1025 to 1150 cm^–1^ due to decreased instrument sensitivity outside this range. The final preprocessed and differenced spectra are shown in Figure S3B.

### PLSR Model Development and Evaluation

The 46 spectra were randomly split into the following subsample sets: the “in-sample” or training set, comprising 19 spectra, used to train the model (i.e., to fit the parameters of the model); the “out-of-sample” or validation set, comprising 13 spectra, used to test the trained model for tuning the model’s hyperparameters (i.e., the number of PLSR components); and the “test-sample” set, comprising the remaining 14 spectra, used to evaluate the final model. Based on *k*-fold cross-validation, the relationship between the root-mean-square error of cross-validation (RMSECV) and the number of PLSR components was observed, revealing that the optimum number of PLSR components to construct the model, that is, where the RMSECV starts to show a marginal decrement, was 3. Finally, the performance of the PLSR model in predicting each compound was evaluated using the root-mean-square error (RMSE) for each compound.

## Results and Discussion

### Mid-IR Spectral Analysis of Pure Compounds

**Figure 2:**
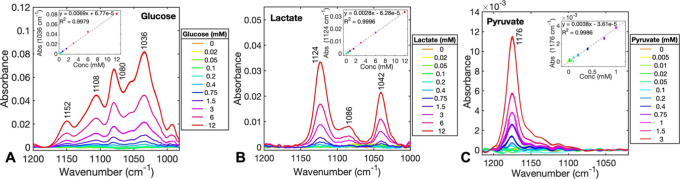
Absorbance spectra of glucose (A), lactate (B), and pyruvate (C) at varying concentrations, measured using QCL-IR transmission spectroscopy with a path-length of 76 μm and an average of 80 measurements. The insets represent the standard curves, including all three repeats, at 1036 cm^–1^ for glucose, 1124 cm^–1^ for lactate, and 1176 cm^–1^ for pyruvate. The spectra of pure perfusion fluid (0 mM) indicate the noise level.

Figure 2 shows the absorbance spectra of the different glucose, lactate, and pyruvate concentrations. The most distinctive spectral features of glucose within the 1000–1200 cm^–1^ range are the C–O vibrations at 1036 and 1080 cm^–1^ and the C–C vibrations at 1108 and 1152 cm^–1^.^25^ The absorbance spectrum of lactate displayed a group of medium-intensity peaks at 1042 and 1124 cm^–1^ and a low-intensity peak at 1086 cm^–1^, which correspond to the C–O stretching vibrations.^26^ Only one strong pyruvate peak at 1176 cm^–1^ was observed in the analyzed spectral region, corresponding to a C–C vibration. Table 2 summarizes the values obtained from the statistical and regression analysis of each pure compound using conventional FT-IR and QCL-IR spectroscopies. The ANOVA and multiple-comparison tests, performed for the strongest peaks of glucose (1036 cm^–1^), lactate (1124 cm^–1^), and pyruvate (1176 cm^–1^), revealed that the statistically significantly different concentration achieved for all three compounds using the QCL-IR system was 0.2 mM, as illustrated in Figure S4. This is a significant improvement compared to the 1.5 mM achieved for glucose using conventional FT-IR spectroscopy (Figure S5C). The ISCUSflex microdialysis analyzer currently used in neurocritical care has linear ranges specified by the manufacturer (M Dialysis AB) for glucose of 0.1–25 mM, lactate 0.1–12 mM, and pyruvate 0.01–1.5 mM. Typical concentration ranges in TBI patients’ brain microdialysates are: 0.1–6 mM glucose, 0.1–8 mM lactate, and 0.01–0.4 mM pyruvate.^5^ Our requirement for this application is to measure the compounds within their entire physiological range. While the LODs achieved for glucose and lactate are adequate for their analysis within the majority of their physiological range, higher sensitivities are required in future work in order to detect all three compounds, particularly pyruvate, within their entire physiological range. This may be achieved by optimizing the optical path-length and instrumentation, as well as using longer averaging times and spectral processing, which we are currently working toward.

**Table 2:** Results Obtained for Each Compound Using Conventional FT-IR Spectroscopy and QCL-IR Spectroscopy

IR (path-length)	compound	peak (cm^–1^)	*R*^2^	ANOVA (mM)	LOD (mM)	LOQ (mM)
FT-IR (50 μm)	glucose	1036	0.995	1.5	0.9	2.8
QCL-IR (76 μm)	glucose	1036	0.997	0.2	0.5	1.7
	lactate	1124	0.999	0.2	0.2	0.7
	pyruvate	1176	0.998	0.2	0.1	0.3

### Analysis of Patient Samples by ISCUSflex and QCL-IR

**Figure 3:**
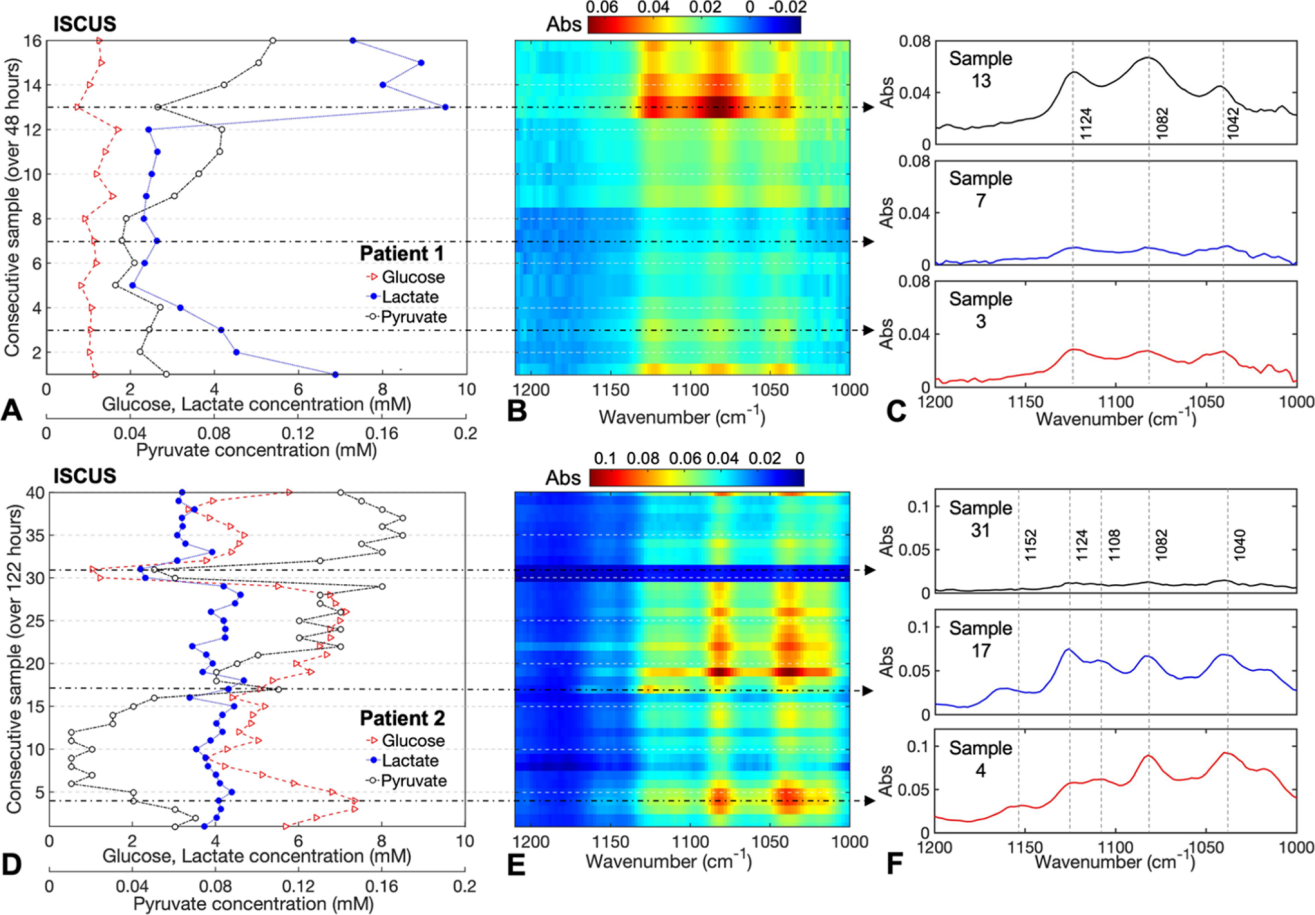
Compound concentrations in consecutively-pooled patient microdialysate samples measured on the ISCUS analyzer for patient 1 over 48 h (A) and for patient 2 over 122 h (D). Corresponding mid-IR spectra acquired on the ChemDetect analyzer for patient 1 (B) and for patient 2 (E). Selected sample spectra illustrating the correlation with ISCUS measurements, as well as the spectral variation between samples collected and pooled at different time-points for patient 1 (C) and patient 2 (F). Note: As a result of sample pooling, each spectrum corresponds to the average concentration within a time-window of 3 to 4 h.

Microdialysate samples were collected from two patients in the neurocritical care unit of Addenbrooke’s Hospital, Cambridge, UK. The samples from each patient were pooled consecutively in order to generate enough sample volume for simultaneous measurements on the ISCUSflex analyzer and ChemDetect analyzer. Therefore, each resulting sample reflects the average concentration over approximately 3 h. Figure 3 shows the correlation between ISCUSflex concentration measurements and QCL-IR spectra for patient 1 (16 samples collected over a period of approximately 48 h, Figure 3A–C) and for patient 2 (40 samples collected over a period of approximately 122 h, Figure 3D–F). The ISCUSflex measurements for patient 1 reveal glucose concentrations between 0.7 and 1.7 mM, lactate concentrations between 2.1 and 9.4 mM, and pyruvate concentrations between 0.03 and 0.10 mM. Absorbance peaks are visible at 1042, 1086, and 1124 cm^–1^ (Figure 3C), corresponding predominantly to lactate absorbance, and are particularly strong for samples 1 and 13–16 (Figure 3B), which correlate with the higher lactate concentrations seen in the ISCUSflex measurements (Figure 3A). The glucose concentrations in this case are particularly low and stable, and therefore glucose absorbance peaks are weak and the changes observed in the spectra correspond predominantly to changes in lactate concentrations. The ISCUSflex measurements for patient 2 reveal glucose concentrations between 1.0 and 7.4 mM, lactate concentrations between 2.2 and 4.7 mM, and pyruvate concentrations between 0.01 and 0.17 mM. In this case, absorbance peaks are predominantly seen at 1040, 1082, 1108, 1124, and 1152 cm^–1^ (Figure 3F), corresponding to a mixture of glucose and lactate peaks, although glucose absorbance peaks are particularly strong for samples 3–5, 19–28, and 40 (Figure 3E), which correlate with the higher glucose concentrations seen in the ISCUSflex measurements for these samples (Figure 3D). The lactate concentrations for patient 2 are relatively stable for all the samples, and therefore the changes in the spectra correspond mostly to changes in glucose concentrations. The sharp decrease in all compound concentrations seen for samples 30 and 31 (Figure 3D) could be due to a sample-collection artifact. Nevertheless, the collected spectra (Figure 3E) show decreased absorbance peaks corresponding to the low concentrations measured by the ISCUSflex. Select sample spectra from each patient, plotted in Figure 3C,F, show a variation of spectral peak intensities at different time-points. Overall, a good correlation was observed between the IR absorbance spectra of the different patient samples over time and the change in concentrations of glucose and lactate determined by the ISCUSflex analyzer.

### PLSR Model Evaluation

The PLSR model was evaluated using the synthetic “test-sample” set and the RMSE values obtained for glucose, lactate, and pyruvate are shown in Table 3. Figure S6 shows the correlation between the predicted versus real concentrations. Based on the clinical requirements, the correlation between the predicted and reference concentrations for glucose and lactate meets acceptable levels of error for clinical implementation. However, this was not the case for pyruvate because it is present at significantly lower concentrations, resulting in low instrument sensitivity and difficulty in observing the pyruvate peak at 1176 cm^–1^ at these concentrations. Therefore, pyruvate was not considered for subsequent analysis in this paper.

**Table 3:** RMSE Values Obtained from the Predicted vs Reference Concentrations of Glucose, Lactate, and Pyruvate for Synthetic Samples and of Glucose and Lactate for Patient Microdialysis Samples

compound	RMSE (mM) synthetic	RMSE (mM) patient 1	RMSE (mM) patient 2
glucose	0.538	0.796	0.335
lactate	0.848	0.809	0.906
pyruvate	0.070		

### PLSR Prediction of Compound Concentrations in Patient Microdialysates

**Figure 4:**
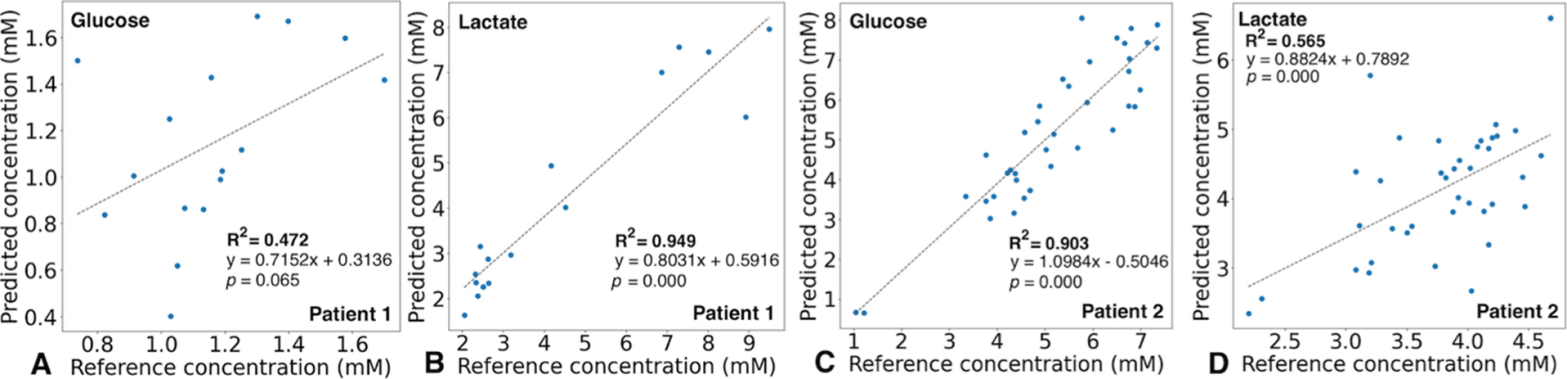
Predicted concentration levels for patient microdialysis samples plotted against ISCUS-measured concentrations for: (A) glucose and (B) lactate for patient 1; and (C) glucose and (D) lactate for patient 2. Dashed lines represent a linear regression fit.

**Figure 5:**
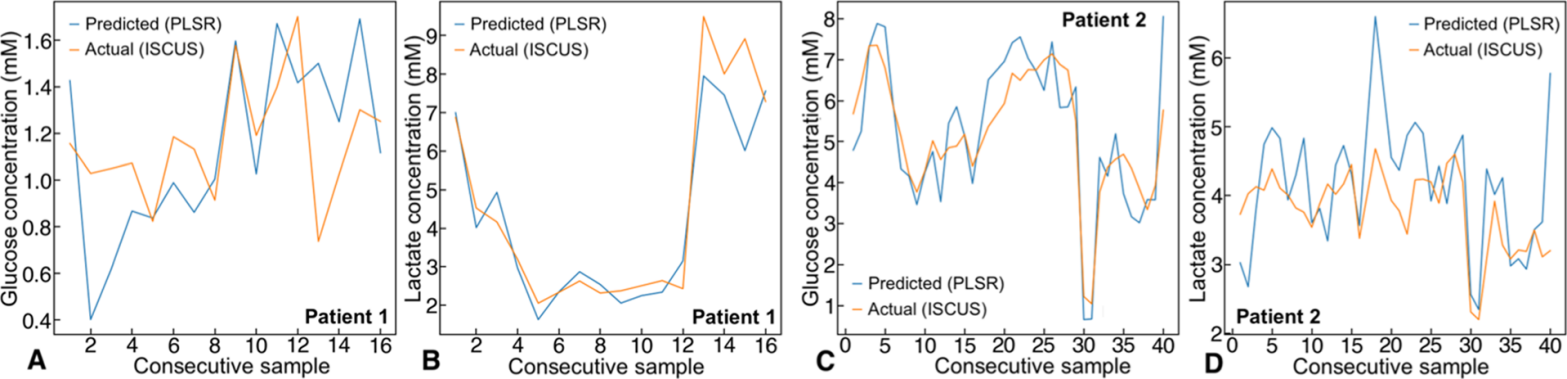
Predicted concentration levels vs concentrations obtained from the standard microdialysis analyzer measured for patient microdialysate samples: (A) glucose and (B) lactate for patient 1; and (C) glucose and (D) lactate for patient 2.

The developed PLSR model was used to compute the concentrations of glucose and lactate in 16 samples for patient 1, and in 40 samples for patient 2. These were compared against reference concentration measurements obtained separately using the gold-standard microdialysis analyzer (ISCUSflex microdialysis analyzer). Figure S7 shows the absorbance spectra obtained for each sample, plotted in the most relevant spectral range, before and after spectral preprocessing. The predicted glucose and lactate concentrations generated by the PLSR model for the microdialysate samples of each patient are shown in Figure 4. The respective RMSE values for each compound are presented in Table 3. These values are comparable to those obtained for the synthetic samples, meaning that the PLSR model built using synthetic samples allows an adequate prediction of patient-sample concentrations, which has the practical implications of potentially not requiring the collection of patient samples for PLSR model development. This may be explained by the fact that microdialysate consists essentially of perfusate enriched with compounds allowed to diffuse through the 100 kDa-cutoff microdialysis catheter membrane. The RMSE obtained for glucose was lowest for patient 2 data, which may be explained by the higher glucose concentration observed compared to lactate, while the RMSE obtained for lactate was lowest for patient 1 data, where the lactate concentrations were higher compared to glucose. Figure 5 shows the concentrations measured using the ISCUSflex versus the PLSR predicted concentrations (from the QCL-IR spectra) for consecutive patient samples over time. The prediction follows the measured concentrations trends effectively, particularly for patient 1 (lactate)—Figure 5B, and patient 2 (glucose)—Figure 5C, also likely due to the reasons mentioned above. This clearly validates the developed PLSR model using synthetic samples for the determination of concentrations of glucose and lactate in TBI-patients' microdialysates. Moreover, it demonstrates the ability of using mid-IR spectroscopy for the continuous monitoring of glucose and lactate at physiological levels, in the brain of TBI patients, over time, which we are presently testing in clinical studies. Currently, the response time for continuous monitoring is limited by the averaging time (approx. 5 min) and the microdialysate flow rate (0.3 μL min^–1^). Our future focus is aimed at reducing the response time down to 5–10 min, which is clinically favorable.

## Conclusions and Outlook

The performance of mid-IR spectroscopy was evaluated as a technique to analyze and monitor the brain chemistry of TBI patients. An LOD of 0.5, 0.2, and 0.1 mM was achieved for glucose, lactate, and pyruvate, respectively, the three main compounds of interest in TBI monitoring. While this allows coverage of most of the physiological range for glucose and lactate, it proved challenging to detect pyruvate, because it is present at much lower concentrations in the brain. A high correlation was seen between the QCL-IR spectra and the compound concentrations obtained from TBI-patients’ cerebral microdialysate samples, measured by the standard ISCUSflex analyzer. The developed PLSR model using synthetic solutions was shown to be promising in predicting the concentrations of the relevant compounds in patient microdialysate samples. This study demonstrated the feasibility of using mid-IR spectroscopy for monitoring the dynamic changes in TBI patients’ brain chemistry over several hours and days. Further work will focus on improving the sensitivity of metabolite detection, particularly for pyruvate, as well as demonstrating clinical measurements of continuous online microdialysis monitoring in TBI patients.

## Supporting Information Available

Image of the QCL-system and integrated flow-cell; histograms summarizing the frequency of observed compound concentrations; absorbance spectra of synthetic samples acquired on a QCL-IR system and preprocessed spectra used for the PLSR model; one-way ANOVA and multicomparison tests; glucose measurements acquired using conventional FT-IR spectroscopy; PLSR model evaluation using the test-sample set; absorbance spectra of patient microdialysates; and list of 50 synthetic sample concentrations for PLSR model development (PDF)
