# Supplementary material for: Cerebral Microdialysate Metabolite Monitoring using Mid-infrared Spectroscopy
Source: Anal Chem. 2021 Aug 25;93(35):11929–36. doi: 10.1021/acs.analchem.1c01149 (PMC8427560; doi:10.1021/acs.analchem.1c01149)
Supplement: Supplementary file 1 — ac1c01149_si_001.pdf [file ac1c01149_si_001.pdf]

Supplementary information

Cerebral Microdialysate Metabolite Monitoring using Mid-infrared Spectroscopy

Farah C. Alimagham,*,‡,† Dan Hutter,§ Núria Marco-García,† Emma Gould,† Victoria H. Highland,‡ Anna Huefner,‡ Susan Giorgi-Coll, † Monica J. Killen, † Agnieszka P. Zakrzewska,† Stephen R. Elliott,‡ Keri L.H. Carpenter,† Peter J. Hutchinson,† Tanya Hutter*,¥,††

‡Department of Chemistry, University of Cambridge, Cambridge CB2 1EW, UK

†Division of Neurosurgery, Department of Clinical Neurosciences, University of Cambridge, Cambridge CB2 0QQ, UK

§Department of Electrical and Computer Engineering, The University of Texas at Austin, Austin TX 78712, USA

¥Materials Science and Engineering Program and Texas Materials Institute, University of Texas at Austin, Austin TX 78712, USA

††Walker Department of Mechanical Engineering, The University of Texas at Austin, Austin TX 78712, USA

DESCRIPTION OF CONTENTS:

Figure S1. Image of the QCL-system and integrated flow-cell.

Figure S2. Histograms summarizing the frequency with which various concentrations of glucose, lactate and pyruvate were observed in the synthetic samples in millimolar (mM) concentrations.

Figure S3. Absorbance spectra of 46 synthetic samples acquired on the QCL-system and pre-processed spectra used for developing the PLSR model.

Figure S4. One-way ANOVA and multi-comparison tests for glucose, lactate and pyruvate.

Figure S5. Glucose measurements using conventional FT-IR spectroscopy.

Figure S6. PLSR model evaluation: correlation between the predicted concentrations for the test-sample set versus real concentrations.

Figure S7. Absorbance spectra of patient microdialysates acquired on the QCL-system.

Table S1. List of 50 synthetic sample concentrations for PLSR model development.

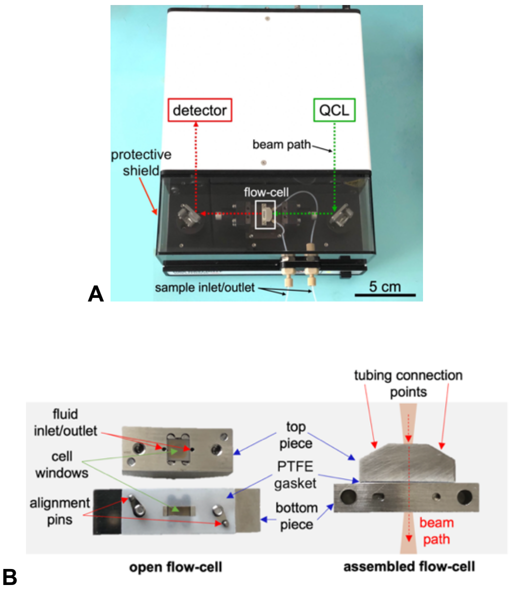

Figure S1. (A) Image of the QCL-system (ChemDetect Analyzer), where the IR light beam (green line) emerges from the QCL and is directed into the sample-containing flow-cell where a portion will be absorbed, after which the remaining (non-absorbed) light (red line) is directed into the detector. Note: the positioning of QCL and detector are not exact; (B) Flow-cell components: "open flow-cell" showing the top and bottom halves of the cell with diamond windows and a fitted PTFE gasket (left side); and "assembled flow-cell" showing tubing connector points and laser-beam path (right side).

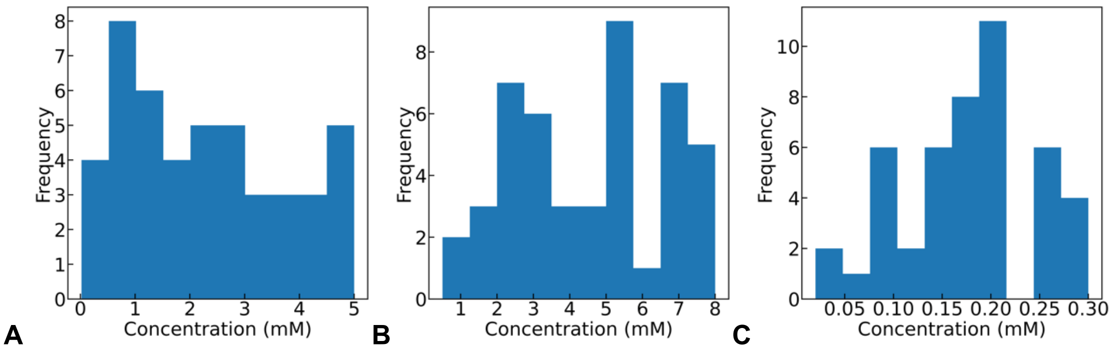

Figure S2. Histograms summarizing the frequency with which various concentrations of (A) glucose, (B) lactate and (C) pyruvate were present in the synthetic samples.

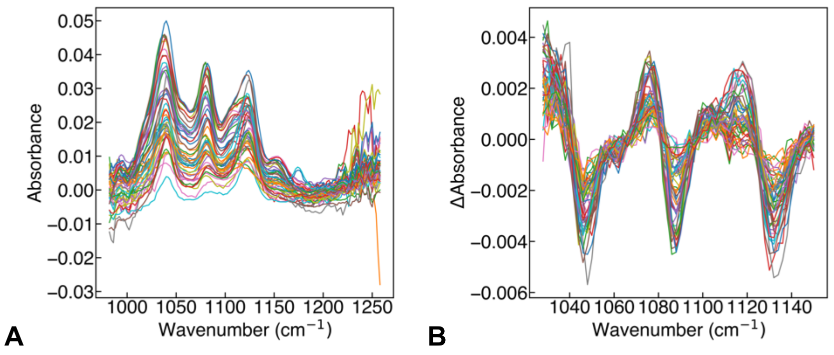

Figure S3. Absorbance spectra of 46 synthetic samples acquired on the QCL-IR system. (A) Spectra excluding outliers; (B) First-order difference spectra with preprocessing applied, used for developing the PLSR model.

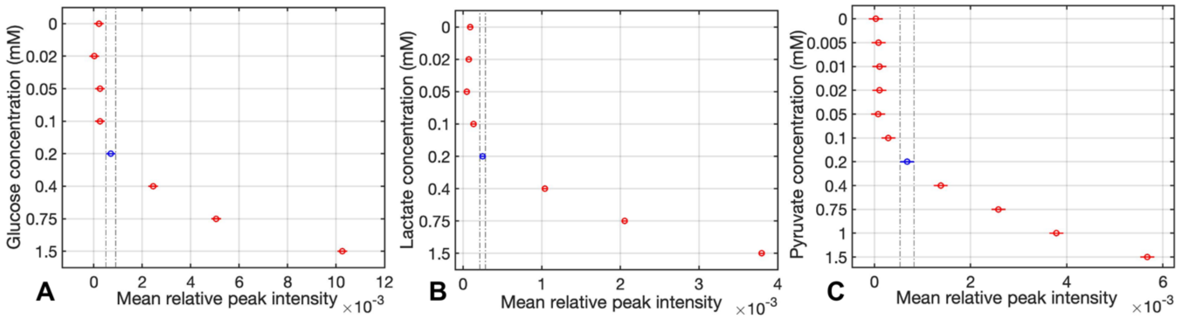

Figure S4. One-way ANOVA and multi-comparison tests at 1036 cm^{-1} for glucose (A), 1124 cm^{-1} for lactate (B) and 1176 cm^{-1} for pyruvate (C). Note: the entire range of concentrations are not shown in the multi-comparison tests for clarity. The statistically significantly different concentrations achieved for all three compounds using the QCL-IR system was 0.2 mM.

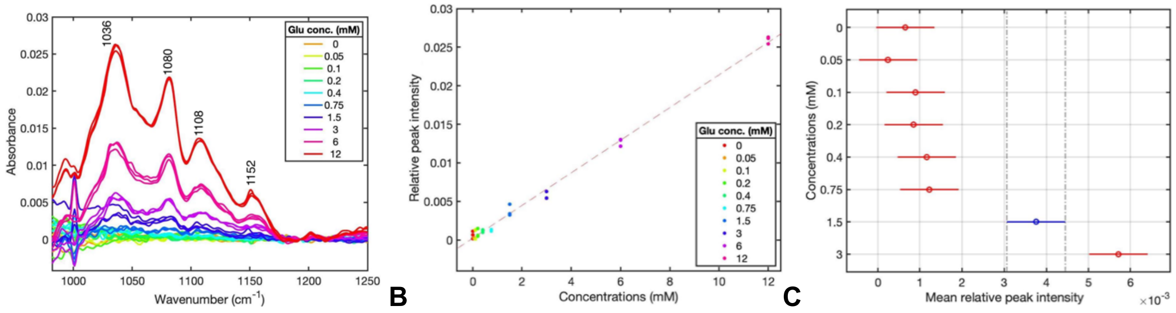

Figure S5. (A) Absorbance spectra of glucose in perfusion fluid at various concentrations, acquired using transmission FT-IR and a 50 µm path-length. Note: spectra normalised to a baseline point at 1180 cm^{-1}; (B) Standard curve of glucose concentrations and corresponding relative peak intensities at 1036 cm^{-1}, with a linear regression fit (R^2 = 0.995, slope = 0.0021); (C) One-way ANOVA and multi-comparison test for the glucose peak at 1036 cm^{-1}. The mean relative peak intensity at 1.5 mM (blue bar) is statistically significantly different to those at all other concentrations (red bars). The calculated LOD and LOQ for the 1036 cm^{-1} glucose peak are 0.9 and 2.8 mM, respectively. Note: the entire range of concentrations is not shown here for simplicity.

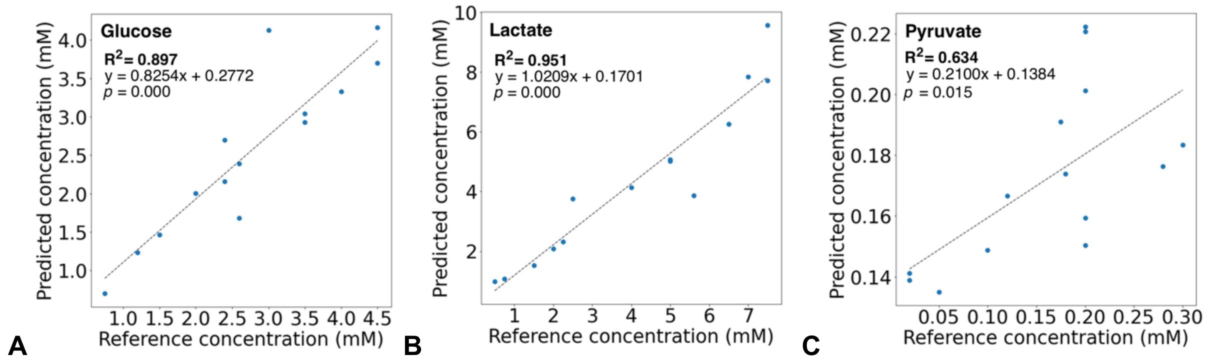

Figure S6. Predicted concentration levels for the 14 test-sample set plotted against reference concentrations for (A) glucose, (B) lactate and (C) pyruvate. Dashed lines represent a linear regression fit.

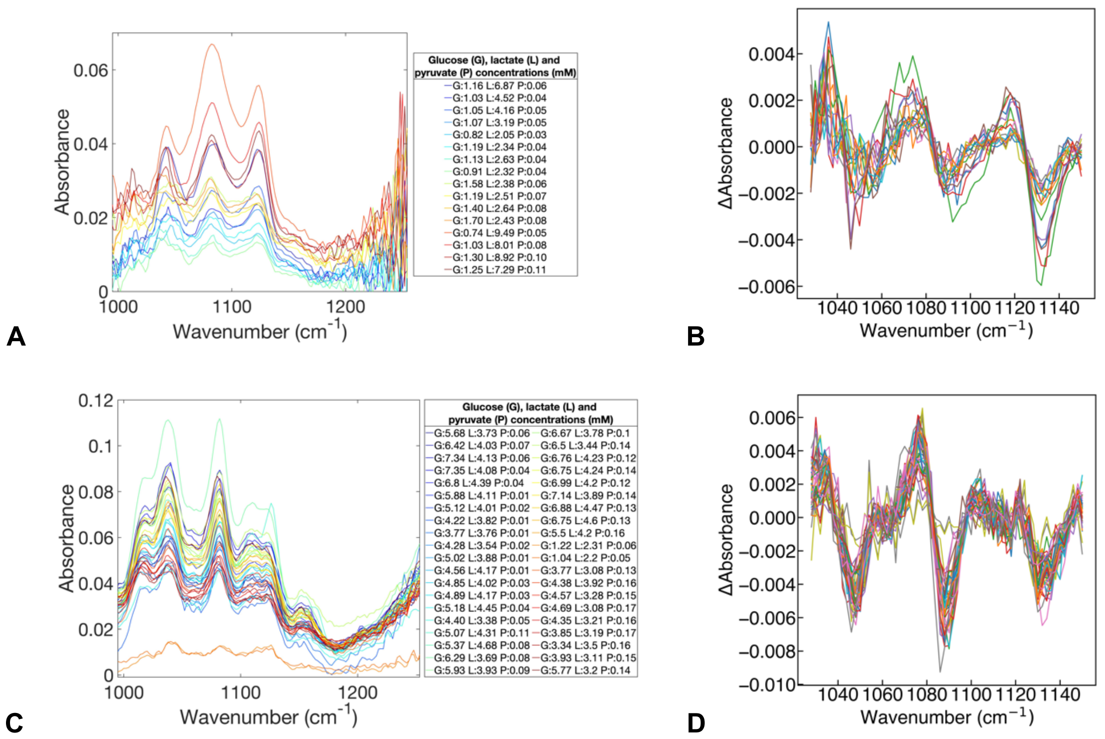

Figure S7. Absorbance spectra of patient microdialysis samples acquired on the QCL system. (A) Patient 1 - Raw Spectra; (B) Patient 1 – First-order difference spectra with preprocessing applied (C) Patient 2 - Raw Spectra; (D) Patient 2 - First-order difference spectra with preprocessing applied.

Table S1. List of 50 synthetic samples with respective concentrations.

Sample no.	Glucose (mM)	Lactate (mM)	Pyruvate (mM)
1	3.6	2.6	0.2
2	2.5	1.75	0.12
3	1.2	3	0.175
4	1.2	7.2	0.28
5	4	6.5	0.175
6	4.8	3.6	0.25
7	0.8	7.8	0.25
8	2.2	3.25	0.08
9	4.5	5	0.1
10	4.8	5.6	0.18
11	4.5	5	0.28
12	1.8	3.25	0.14
13	2.4	3.5	0.2
14	2.4	0.75	0.02
15	3.5	2	0.1
16	0.8	5.6	0.2
17	4	5	0.14
18	4	7.2	0.25
19	0.12	6.5	0.25
20	1.8	2.5	0.1
21	2	2.8	0.14
22	0.02	3.25	0.2
23	2.6	4	0.12
24	0.75	2.25	0.2
25	3	2.5	0.2
26	4.5	2.5	0.2
27	3.5	1.5	0.05
28	0.55	5.6	0.28
29	4.8	1.75	0.08
30	1	4	0.3
31	2.2	7.5	0.2
32	4	4.5	0.1
33	0.7	3.25	0.25
34	1.2	7	0.2
35	0.8	6.5	0.175
36	0.8	4.5	0.25
37	5	4	0.08
38	3	5.6	0.15
39	1.2	5	0.1
40	2.5	6.5	0.14
41	0.5	2.5	0.2
42	2.5	5.6	0.175
43	1.5	5	0.28
44	1.5	8	0.175
45	4.8	7.5	0.14
46	2	0.5	0.02
47	0.45	6	0.175
48	0.65	7	0.25
49	3.5	4.5	0.18
50	4.4	7.5	0.2
